# Detection and monitoring of hypermethylated *RASSF1A* in serum from patients with metastatic breast cancer

**DOI:** 10.1186/s13148-016-0199-0

**Published:** 2016-04-01

**Authors:** Søren Kristiansen, Dorte Nielsen, György Sölétormos

**Affiliations:** Department of Clinical Biochemistry, Nordsjællands Hospital–Hillerød, University of Copenhagen, Dyrehavevej 29, DK-3400 Hillerød, Denmark; Department of Oncology, Herlev Hospital, University of Copenhagen, Herlev Ringvej 75, 2730 Herlev, Denmark

**Keywords:** DNA methylation, Epigenetics, Monitoring, Metastatic breast cancer, *RASSF1A*

## Abstract

**Background:**

Circulating hypermethylated *RASSF1A* could be a novel and potential useful marker for monitoring patients with metastatic breast cancer. Technical obstacles include fragmentation of the circulating DNA, fluctuations in the concentration, low concentrations of circulating tumor DNA, and different locations of methylation in the *RASSF1A* gene among patients. One common method for detection of hypermethylated genes is sodium bisulfite conversion of non-methylated cytosine to uracil, followed by detection with PCR. However, the method relies on full conversion of all non-methylated cytosines, cause strand breaks, and loss of DNA. Alternatively, methylation-sensitive restriction enzymes have been used to digest genomic DNA, as well as sodium bisulfite-treated DNA. By flanking different regions of the *RASSF1A* with different PCR primer pairs, we analyzed for methylated genomic regions resistant to cleavage by the methylation-sensitive restriction enzymes HpaII and BstUI. The goal was to find region(s) in *RASSF1A* with high sensitivity and specificity that could be used for monitoring.

**Results:**

The serum was spiked with non-human control DNA. By tracing the spiking control, the isolation procedure of the rare circulating tumor DNA was initially optimized. By analysis of production of PCR amplicons from HpaII- or BstUI-treated DNA isolated from 24 patients with metastatic breast cancer, we located four regions resulting in sensitivities from 63 to 83 %. When examining samples from 24 control subjects, these four regions gave a specificity of 100 %. Among these four regions, the primer pair with the highest PCR efficacy was selected to monitor the *RASSF1A* concentration in 31 collected serum samples. The spiked DNA was then used to calculate the tumor *RASSF1A* concentrations independent of fluctuations in circulating non-tumor DNA. As a proof of principle, there was concordance in the kinetics of the *RASSF1A* and the serological cancer biomarkers CA 15-3, CEA, and TPA.

**Conclusions:**

Methylation-sensitive restriction enzymes may be a useful methodological approach for monitoring circulating hypermethylated *RASSF1A* among patients with metastatic breast cancer.

## Background

The clinical oncologist must assess and balance multiple information obtained from physical examinations, imaging techniques, a broad pallet of biochemical laboratory tests in blood, as well as pathological examinations of biopsies to make decisions whether the disease is under control and the adverse effects are acceptable [1].

Among patients with metastatic breast cancer, serological tumor markers alone are not recommended for monitoring the response to anticancer therapy [1]. An area of development of a new generation of blood-based cancer biomarkers for monitoring breast cancer patients could be hypermethylated tumor suppressor genes [2]. The cytosine in the genes is methylated by endogenous DNA methyltransferases with affinity for CpG dinucleotide motifs. The CpG motifs are condensed in CpG islands in the promoter regions, which are progressively more densely methylated during tumorigenesis. One method for detection of hypermethylated genes is sodium bisulfite conversion of non-methylated cytosine to uracil [3]. Since methylated cytosine is protected from conversion, primers and probes can then be designed to target hypermethylated genes, control genes, or regions not susceptible to methylation by PCR amplification of converted DNA [4]. However, the sodium bisulfite conversion of the DNA may be incomplete [5] and cause breaks in the DNA strands [6] and critical loss of total sample DNA [7, 8]. These critical issues may compromise the analytical detection of hypermethylated genes.

One candidate gene for development of a novel biomarker could be the RAS association (RalGDS/AF-6) domain family member 1A (*RASSF1A*) gene [9]. Close examination of the methylation status of *RASSF1A* reveals that hypermethylation occurs in the promoter and exon 1 region in a large percentage of human breast cancers [10], but the distinct location of the methylated CpGs are differentially distributed among different tumors [11]. Methylation profiling of individual tumors further suggests that DNA methylation progressively spreads from the first exon into the promoter area of the *RASSF1A* gene [11]. Furthermore, wide clonal intra-tumor heterogeneity of promoter hypermethylation of *RASSF1A* has been found in breast cancer [12]. Thus, there are both intra- and interindividual differences in the methylation profiles of the *RASSF1A* gene among tumors from different patients. Thus, one primer and probe design may not cover all types of *RASSF1A* methylation profiles, and the patient-to-patient variation may decrease the clinical sensitivity.

In a previous study, we used sodium bisulfite-converted DNA and MethyLight to quantitate changes in serial concentrations of the hypermethylated *RASSF1A* in serum [13]. Serum samples were obtained from 29 patients with advanced breast cancer undergoing therapy and from 18 healthy control subjects [13]. Hypermethylated *RASSF1A* was not detected in serum samples obtained from the healthy women but was detected in all of the 29 patients at some time during monitoring. Hypermethylated *RASSF1A* was detected in a total of 45 % of the 422 serial patient samples. Thus, *RASSF1A* was only periodically detected in some patients during monitoring. Furthermore, the collagen 2 A1 gene was used as a marker for the non-tumoral DNA concentration in the serum sample primers and used to normalize the *RASSF1A* concentration. However, the non-tumoral DNA concentration may not be constant in the blood due to a wide variety of confounding factors [14–17]. The acute effect of therapy may also cause a burst of DNA into the blood [18]. Thus, normalization of circulating tumor DNA to non-tumoral DNA may give false information regarding the status of the disease. Thus, bisulfite conversion [5–8], normalization of tumoral DNA to non-tumoral DNA [14–18], and one probe/primer design [10–12] may not be an optimal method for monitoring hypermethylated DNA among patients with metastatic breast cancer, as shown in the previous study [13].

In the present study, we have omitted the sodium bisulfite treatment and changed the methodological procedure. Firstly, we spiked the serum samples with external DNA. The spiking control was used to (1) optimize the DNA isolation steps and (2) to express the concentration of *RASSF1A* in nmol/ml. Thus, the concentration of hypermethylated *RASSF1A* was not depending on the fluctuating non-tumoral control DNA. Secondly, we used the restriction enzymes HpaII and BstUI to cleave unmethylated 5′-CCGG and 5′-CGCG motifs in the DNA to detect uncleaved hypermethylated *RASSF1A*. Thirdly, we also tested different primer pairs flanking different DNA regions of the *RASSF1A* gene to encounter for the intra- and interbiological variation of the methylation profile to ensure optimal sensitivity and specificity. Finally, as a proof of principle, the primer pair with the highest clinical sensitivity, specificity, and PCR performance were used to monitor serial changes in the concentrations of *RASSF1A* in serum obtained from a patient with metastatic breast cancer during therapy.

## Results

Serum samples were obtained from 24 patients with metastatic breast cancer and from 24 age-matched healthy female as controls. The average concentrations of the protein tumor markers cancer-antigen 15-3 (CA 15-3), carcinoembryonic antigen (CEA), and tissue polypeptide antigen (TPA) among patients and healthy females are shown in Table 1. The concentrations spanned a wide range. The concentrations of CA 15-3, CEA, and TPA were significantly higher in serum from breast cancer patients as compared to the healthy females. In a few patients, the marker concentrations were below the cut of level. One sample had a CEA concentrations <7.5 μg/ml and two samples had a TPA concentration <357 U/L, whereas all CA 15-3 concentrations were >30 kU/L. All the healthy females had CA 15-3, CEA, and TPA concentrations below the respective cut of levels.

**Table 1:** Protein tumor marker concentrations among the investigated patients with metastatic breast cancer and healthy females

	CA 15-3 (mean ± SE, *n* = 24)	CEA (mean ± SE, *n* = 24)	TPA (mean ± SE, *n* = 24)
Patients with metastatic breast cancer	1552 ± 384 kU/L^a^	559.7 ± 177 μg/L^a^	2639 ± 657 U/L^a^
Age-matched healthy females	14.4 ± 1.1 kU/L	3.1 ± 0.3 μg/L	56.8 ± 10.8 U/L

^a^Significantly different from age-matched control concentration (*p* < 0.05). The serum samples were used to isolate DNA, which was used to calculate the sensitivity and specificity of six different primer designs against hypermethylated *RASSF1A*

Six different primer pairs were designed against *RASSF1A* promoter region and exon 1 (NG_02327.1, region 4562-5517). The primers targeted different locations of the gene and targeted different numbers of HpaII and BstUI cleaving motifs (Table 2). The investigated *RASSF1A* sequence contained 88 CpG dinucleotide sites, which may be aberrantly methylated as a response to neoplasia. Among these sites, there are seven HpaII and sixteen BstUI sites. The six designed primer pairs covered six HpaII and fourteen BstUI sites, covering 39 % of all CpG sites. The base pair length of the designs was kept below <300 bp in order to maximize the chance of amplifying fragmented DNA [16].

**Table 2:**
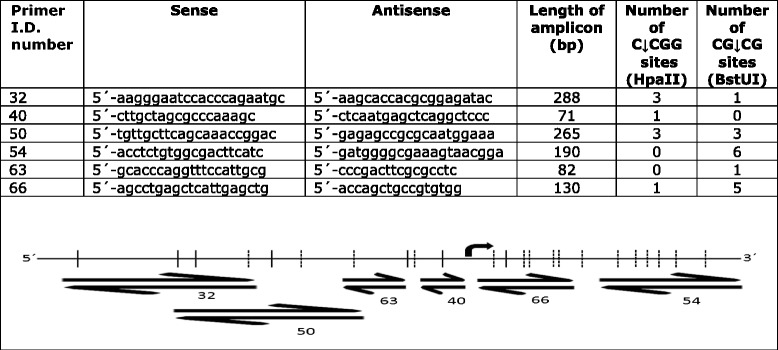
Overview of the methylation profile of *RASSF1A*

The sequences of six different primer pairs designed against *RASSF1A*, the length of the amplicon, and the number of C↓CGG and CG↓CG sites for each primer pair are shown in the upper panel

The lower panel shows the location of C↓CGG (*solid line*) and CG↓CG (*broken line*) cleavage sites in *RASSF1A*. The primer pairs were designed to target these C↓CGG and CG↓CG sites. Methylation of the cytosine in the CpG dinucleotide motif within the C↓CGG and CG↓CG sites protects against enzymatic restriction cutting by HpaII and BstUI, respectively, and enables formation of amplicons. Unmethylation results in digestion of the target and no formation of amplicons. The location of the initiation site by the codon ATG is show by an arrow

**Fig. 1:**
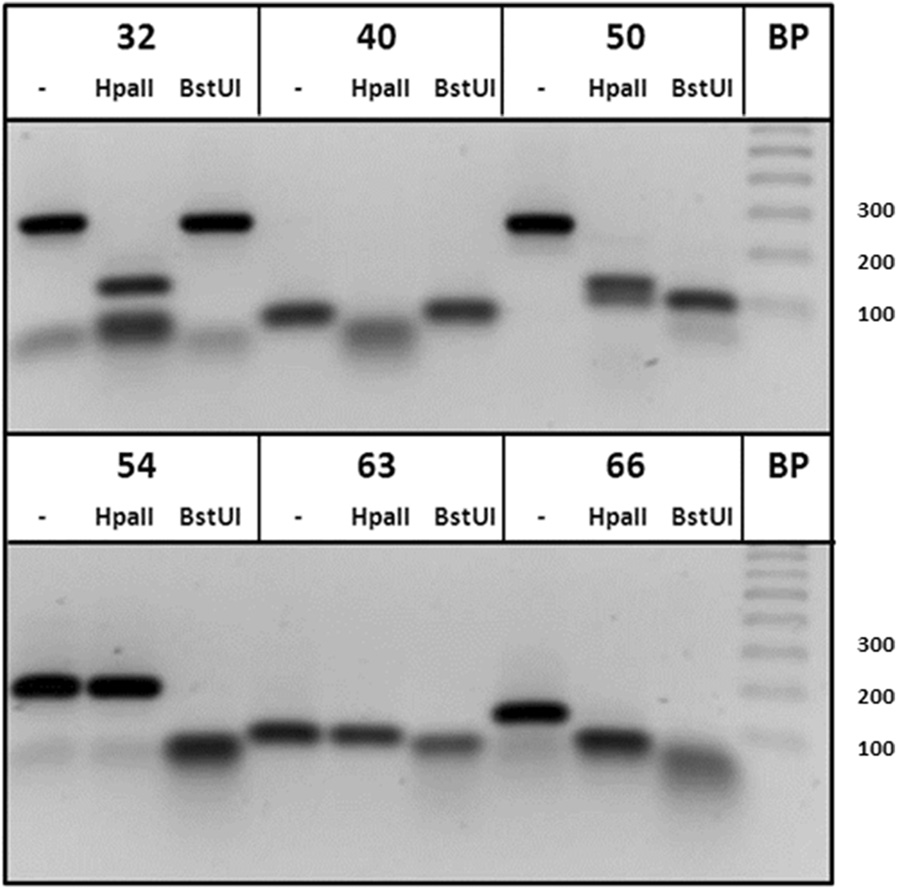
HpaII and BstUI restriction fragments. PCR amplicons produced with primer set 32, 40, 50, 54, 63, and 60 were incubated with HpaII or BstUI restriction enzymes, or mock-digested (−). Since PCR amplicons are not methylated, all HpaII and BstUI motifs sites are cleaved. The DNA fragments were separated on an agarose gel and UV-illuminated by ethidium bromide staining. Amplicons produced by primer pair 40 was exclusively cleaved by HpaII; primers 54 and 63 were only cleaved by BstUI; and the amplicons produced by 32, 50, and 66 were cleaved by both HpaII and BstUI. The BstUI cleavage of the amplicon produced by primer pair 32 was calculated to reduce the length with 9 bp. This minor reduction in length may not be visually detectable by agarose gel electrophoresis

To validate the six primer pairs, cDNA amplicons were produced and mock-digested or incubated with HpaII alone, or BstUI alone, respectively. Since PCR amplicons are not methylated, all HpaII or BstUI cleavage motifs will be cleaved in the amplicons. The digest was separated on 1× TBE ethidium-stained agarose gels, as shown in Fig. 1. Primer pairs designed to contain exclusively HpaII cleavage sites, BstUI sites, or both type of cleavage sites, displayed a digestion profile as expected. However, the 288-bp amplicon produced with primer 32 was calculated to be reduced to 279 bp after BstUI cleavage. This minor reduction in base pair length was not visually detectable by agarose-electrophoresis.

**Fig. 2:**
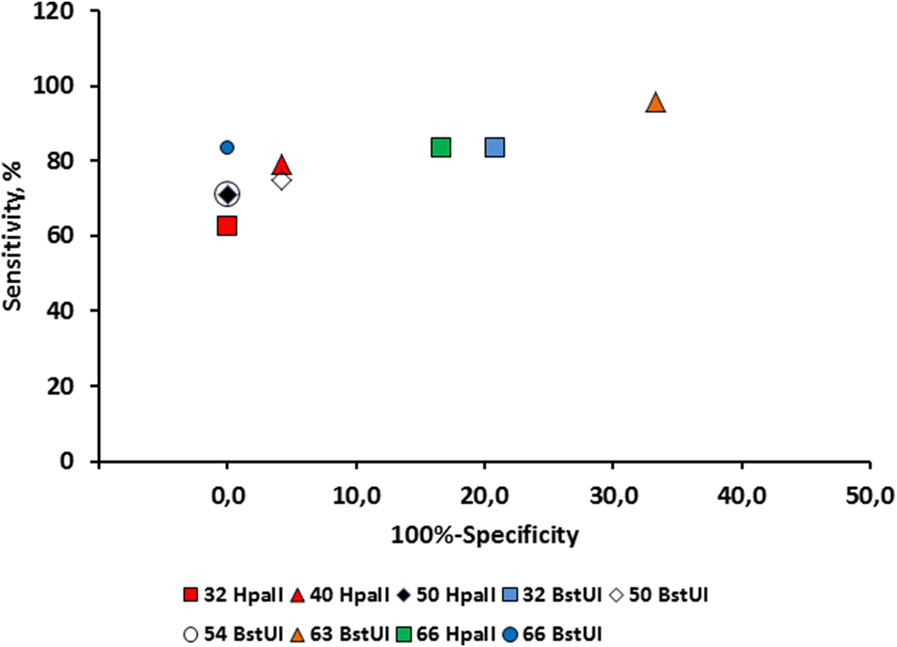
ROC curve for detection of hypermethylated *RASSF1A.* Data plot of the sensitivity and 100 % specificity. Serum DNA was isolated from serum from 24 patients with metastatic breast cancer and 24 age-matched healthy control individuals. The DNA was incubated with either the methylation-resistant restriction enzymes HpaII or BstUI and thereafter PCR amplified with six different primer designs (primer ID # 32, 40, 50, 54, 63, and 66, as explained in Table 2) covering different regions of the promoter and exon 1 of *RASSF1A*

Productions of amplicons from HpaII- or BstUI-treated serum DNA from 24 metastatic breast cancer patients and 24 healthy controls were then used to calculate the sensitivity and specificity. This was plotted in a receiver operating characteristic (ROC) curve, as shown in Fig. 2. When DNA was BstUI-treated and subsequently PCR amplified with primer pair 66, amplicons was detected in 20 of 24 serum samples (83 % sensitivity) isolated from metastatic breast cancer patients. In contrast, no amplicons were produced when using DNA isolated from serum obtained from 24 healthy females (100 % specificity). This design targeting five BstUI sites distal to the initiation site of *RASSF1A* gave the highest AUC in the ROC plot relative to the eight other primer combinations. Primer 50 HpaII, 54 BstUI, and 32 HpAII had a clinical specificity of 100 %, but the sensitivity was relatively lower, 71, 71, and 63 %, respectively.

**Fig. 3:**
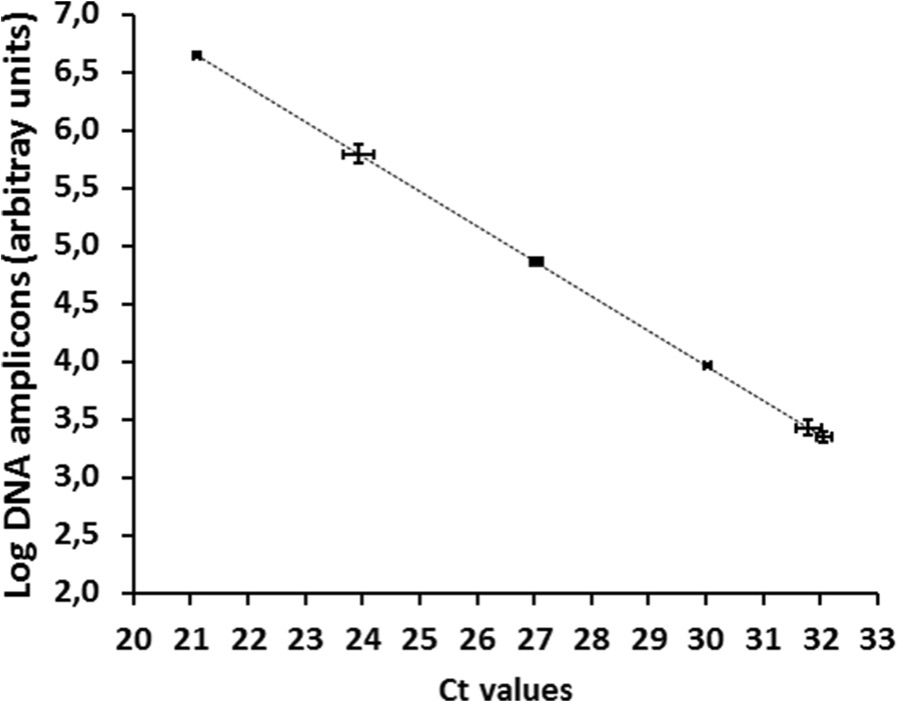
Correlation of the Ct values and logarithmic DNA amplicons*.* DNA was diluted sequentially tenfold and PCR amplified with primer no. 50. Each measurement was performed as triplicates (mean ± SD). Ct (cycle threshold) is defined as the number of cycles required for the fluorescent signal to cross the threshold

Since monitoring require precise measurements of the serial concentrations of rare tumor DNA, the four primer pairs with 100 % clinical specificity were then tested for their analytical imprecision in terms of coefficient of variation, PCR amplification factor, efficiency, linearity, and limits of detection. As shown in Fig. 3, PCR amplification with primer 50 developed a signal linear over a 10^6^-fold change in DNA concentration, an amplification factor of 1.98 and efficiency of 98.02 %. The analytical coefficient of variation was 13.2 % (*n* = 30 determinations), and the limit of detection was 0.01 ng/ml of DNA. Overall, primer 50 was marginally superior to the three other primer pairs and was selected to amplify HpaII-treated DNA isolated from the serum samples.

**Fig. 4:**
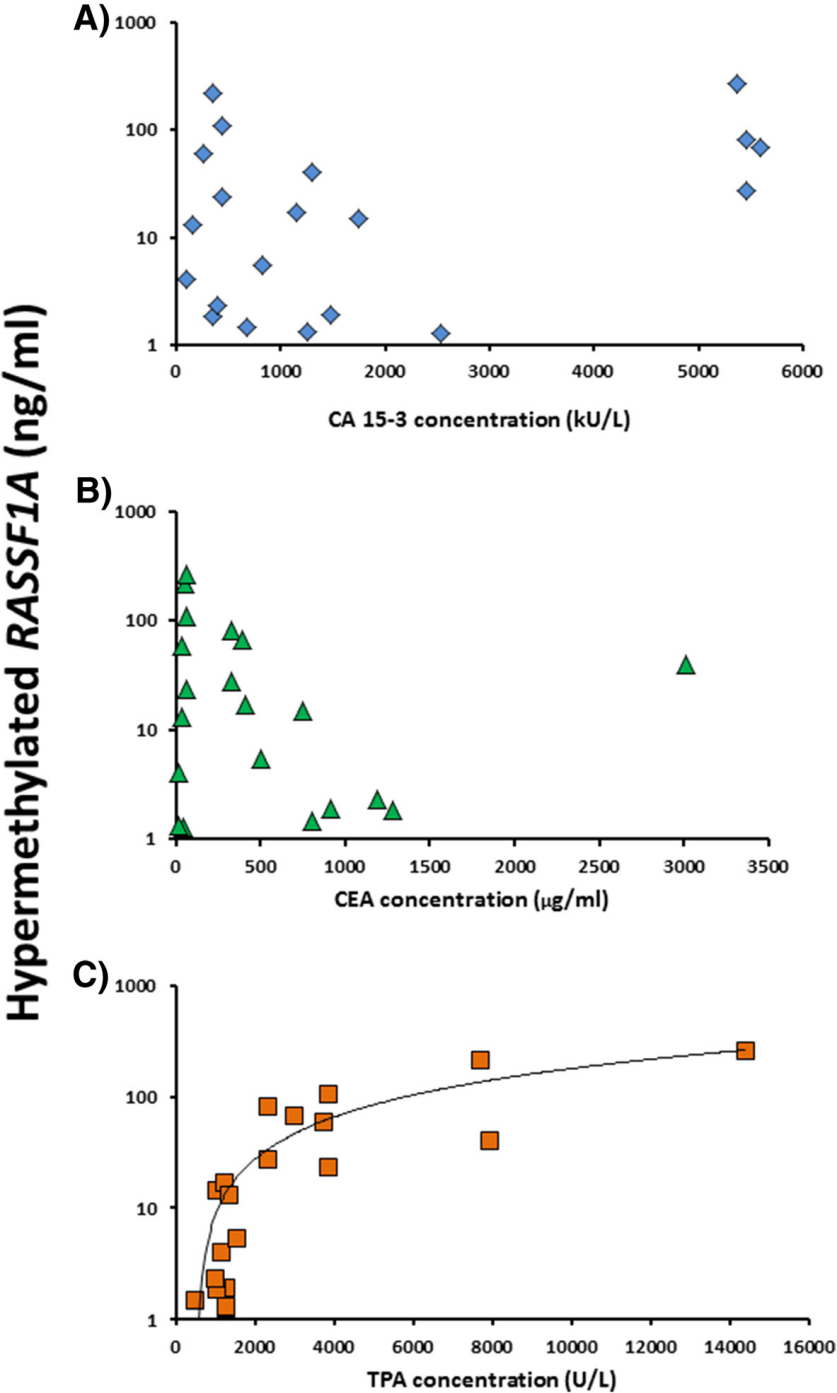
Correlation between the concentrations of the protein tumor markers and hypermethylated *RASSF1A* based on the cross-sectional samples from each of the 24 patients. The serum concentration of CA 15-3 (**a**), CEA (**b**), and TPA (**c**) are plotted against the average hypermethylated *RASSF1A* concentration determined by using all six primer designs. The data was obtained from 24 serum samples collected from patients with metastatic breast cancer

The average concentration of the hypermethylated *RASSF1A* using all primer designs was plotted against the serum concentrations of the tumor burden markers CA 15-3 and CEA and the tumor activity marker TPA. As shown in Fig. 4, there was a concordance of the concentrations of TPA with the concentrations of the hypermethylated *RASSF1A*. There was discordance of the CA-15-3 and CEA concentrations with the hypermethylated *RASSF1A* concentrations.

**Fig. 5:**
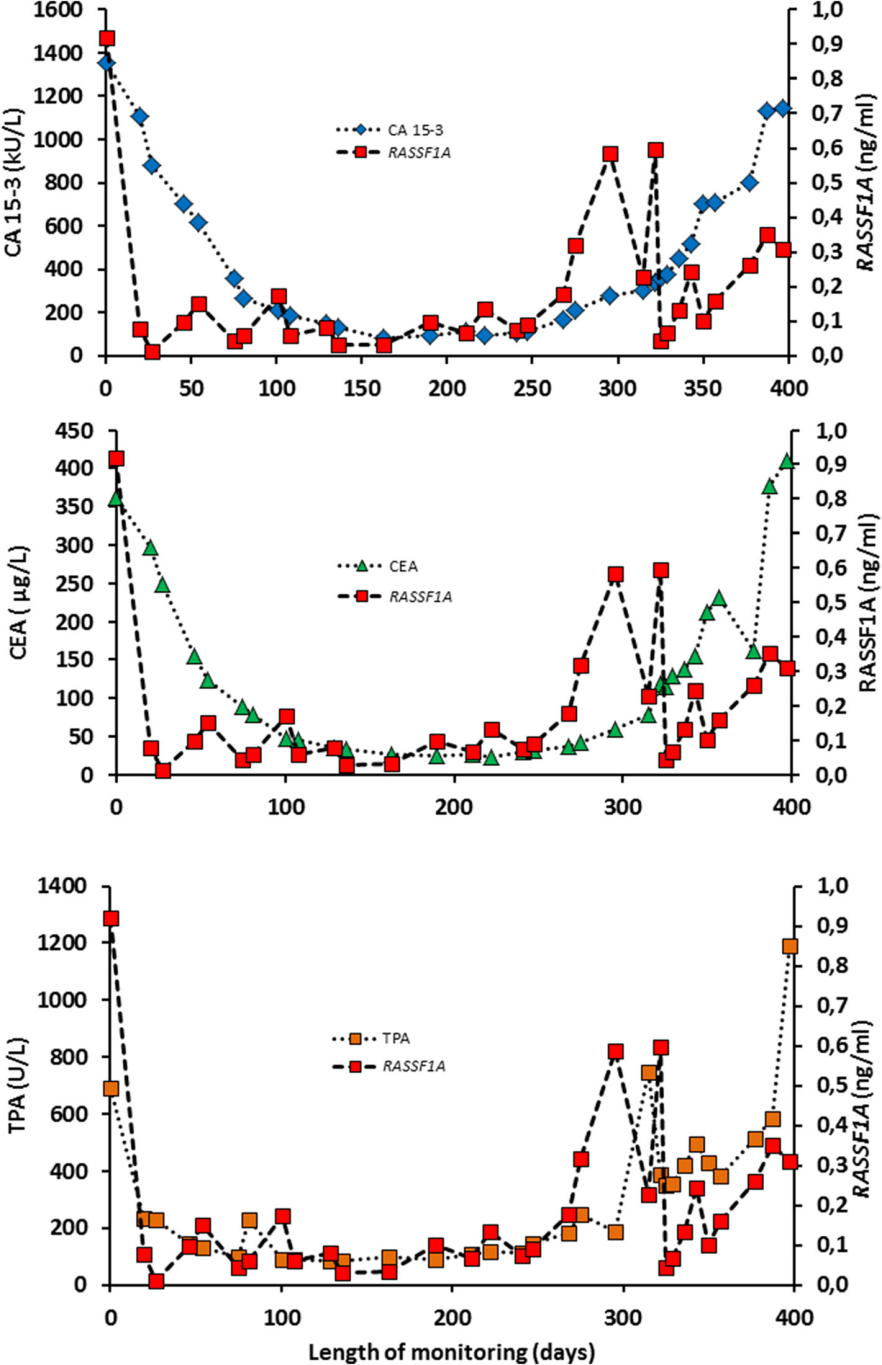
Monitoring of patient A with metastatic breast cancer during therapy. Three panels with serial concentrations of the protein biomarkers CA 15-3, CEA, TPA and the concentrations of hypermethylated *RASSF1A*

As the first proof of principle, 31 independent serial data for the tumor markers CA 15-3, CEA, TPA and hypermethylated *RASSF1A* for one patient A is shown in Fig. 5. Overall, there was concordance with the change in tumor marker concentrations and the change in *RASSF1A* concentrations.

## Discussion

Hypermethylation of the *RASSF1A* gene is a prime candidate among many genes as a potential novel biomarker for stage I–IV breast cancer and have been investigated for early detection of cancer, diagnosis, prognosis for the patient, prediction of the effect of therapy, and monitoring of the effect of therapy by measuring the circulating biomarker concentration (reviewed in [9]). In a previous study, we monitored circulating hypermethylated *RASSF1A* and showed concordance with the kinetics of CA 15-3, CEA, and TPA but also the periodical lack of *RASSF1A* detection in some patients with advanced breast cancer [13]. The periodical lack of detection could be due to in situ sub-detectable concentrations and/or due to an analytical problem with sodium bisulfite conversion of fragmented DNA [13].

In the present study, we have optimized the steps for isolating the rare serological tumor DNA fragments by tracing a spiking control. Instead of using sodium bisulfite treatment of DNA, we used restriction enzymes; these are sensitive to methylation of the CpG motif in the cleavage site. By using restriction enzyme-treated as DNA template, flanking primer pairs were used to amplify different locations of *RASSF1A.* The different designs were tested for optimal sensitivity, specificity, and PCR performance. With this method, we found a sensitivity of 63–83 % at a specificity of 100 % when testing 24 cross-sectional samples collected from metastatic breast patients and healthy females.

In the four previous cross-sectional studies of sodium bisulfite-converted DNA, the circulating hypermethylated *RASSF1A* was detected in metastatic breast cancer patients [19–22]. Tan et al. [19] detected *RASSF1A* in 42 % (8 out of 19) of the serum samples with a technique based on methylation-specific PCR of sodium bisulfite-converted DNA. The other studies [20, 21] both used the MethyLight PCR technique but with different primers and probes raised against sodium bisulfite-converted DNA. Matuschek et al. [20] detected *RASSF1A* in 43 % (10 out of 23) of the serum samples, whereas Van der Auwera et al. [21] detected 35 % (28 out of 79). Fackler et al. [22] used the cMethDNA assay to determine the concentrations of ten individual genes. The assay is depending on the input of sodium bisulfite-converted DNA, which are amplified in two sequential PCR reactions; first, multiplex PCR amplification of the ten genes with external primers, then followed by quantification of individual genes by real-time methylation-specific PCR reaction. Detection of *RASSF1A* alone was achieved in 70 % (40 out of 57) of the training and test samples, but the sensitivity was raised to 91 % at a specificity of 96 % when combining the panel of genes. Taken together, in these studies using different methods, but bisulfite-converted DNA, the sensitivity was in the range of 35–70 % [19–22]. In the present study, using input of genomic DNA, two different methylation-sensitive restriction enzymes, the sensitivity was 63–80 % at a specificity of 100 % for four different regions of *RASSF1A*. In contrast, the low sensitivities for detection of *RASSF1A* in the other studies could be due sodium bisulfite conversion of DNA, which may limit the amount of the rare tumor DNA [19–21, 23]. Thus, detection of genes in a panel with methylation-sensitive restriction enzyme-digested DNA as assay input should be further investigated.

The large span and variations in the CA 15-3, CEA, and TPA concentrations among the cross-sectional samples were used to test for correlation between the protein biomarkers and hypermethylated *RASSF1A* concentrations (Fig. 4). The CA 15-3 assay is based on the monoclonal antibodies 115D8 and DF3 which are both raised against the human MUC1 protein. The CEA molecule is a glycoprotein involved in cell adhesion; it is a glycosylphosphatidylinositol cell surface anchored glycoprotein that is released in to the bloodstream of cancer patients and healthy individuals. Being a secretory product, both CA 15-3 and CEA are considered as serological markers of changing tumor burden in the individual patient. TPA belongs to the cytoskeleton proteins circulating as a complex of soluble proteolytic polypeptide fragments of cytokeratins 8, 18, and 19. The TPA release may indicate cell turnover and the information supplied by the TPA may be distinctly different from the information supplied by the markers of tumor burden CA 15-3 and CEA [24]. As shown in Fig. 4, the present preliminary results may suggest that circulating hypermethylated *RASSF1A* may be a biomarker for cell turnover and/or cell death by apoptosis/necrosis. However, as shown in Fig. 5, we found concordance with hypermethylated *RASSF1A* with the kinetics of CA 15-3, CEA, and TPA. This may be due to the similar kinetics of the three markers CA 15-3, CEA, and TPA.

Monitoring of the changes in serial biomarker concentration requires an analytical method with a low-as-possible analytical coefficient of variation [25]. When the concentration of circulating tumor DNA is expressed relatively to the circulating non-tumor DNA, the change in concentration may be misleading. In the present study, we used a spiking control to account for small pre-analytical variations in the DNA concentrations. Spiking of serum samples has previously been used by Fackler et al. [22] to facilitate quantification of tumor DNA. The optimal location in the *RASSF1A* gene and PCR performance were then used to monitor metastatic breast cancer patients during therapy. As a proof of principle, 31 serial samples were monitored with the tumor markers CA 15-3, CEA, and TPA and compared with the changes in the hypermethylated *RASSF1A* concentrations. In the present study, and in the previous study [13], concordance could be observed between serial changes of the hypermethylated *RASSF1A* gene and the protein markers.

## Conclusion

In conclusion, it is suggested to use methylation-sensitive restrictive enzymes as an alternative to the time-consuming sodium bisulfite step, which also may reduce the amount of tumor DNA. Circulating hypermethylated *RASSF1A* and protein markers may have similar kinetics during monitoring. Future studies are required to define criteria for changes in tumor DNA to assess the disease status. These criteria should be used to explore the clinical utility of monitoring circulating *RASSF1A*.

## Methods

### Healthy subjects

Women among the healthy staff at the Departments of Oncology and Clinical Chemistry, Herlev Hospital, University of Copenhagen, Denmark, volunteered to participate in the study from 1990 to 1992 [26]. All subjects gave informed consent to their participation, and the study was approved by the regional Ethical Committee (KA 93076). All subjects stated they were free of disease at the time of the study, and none had any known chronic or recurrent illness or was taking any medication. The subjects continued their usual lifestyle during the period of the study. No investigations were performed to exclude asymptomatic breast cancer. Serum samples was stored at −80 °C and later used for detection of hypermethylated *RASSF1A*.

### Patients with metastatic breast cancer

The investigated patients had histologically proven advanced progressive breast cancer (stage IV) with measurable or evaluable disease [26, 27]. They received epirubicin 70 mg/m^2^ on days 1 and 8 every 4 weeks. Epirubicin was continued until progressive disease (PD) was noted or until a maximum cumulative dose of 1000 mg/m^2^ had been administered. Clinical response evaluations at that time of the study (1988 to 1991) were based on the criteria of the World Health Organization [28] and performed by investigators without knowledge of the tumor marker data. Blood specimens for CA 15-3, CEA, and TPA analysis were sampled before each treatment cycle [26, 27]. At each sampling, the serum specimen used for analysis of the protein tumor markers was saved in different aliquots at −80 °C and used for the current analysis of hypermethylated DNA. The study complied with the Helsinki II Declaration and was approved by the Scientific Ethics Committee of Copenhagen County (KA 89257, H-D-2009-048).

### Isolation of tumor DNA

One milliliter of serum from the bio bank was quick-thawed and spiked with a constant amount of PBR322 plasmid (Life Technologies). The spiking control was traced during all the isolation steps. The spiked serum was immediately transferred to 1 ml of binding buffer supplemented with RNA carrier. The DNA isolation was carried out as suggested by Roche Diagnostics (High Pure Viral Nucleic Acid Large Volume Kit), except that the DNA and tracer were eluted with 0.1 ml of Tango buffer (Fisher Scientific). The DNA capturing efficiency of column filters was investigated by repassing the sample DNA on the columns. One DNA capturing cycle was found sufficient (data not shown). The eluting efficiency of the captured DNA from the filters was investigated by eluting filters with different volumes of elute buffer (buffer suggested by the manufacturer), water, or Tango buffer. The optimal method was to elute the DNA from filters with 100 μl of Tango buffer. There was no difference between the recovery and relative concentration of human DNA versus the spiking control plasmid. Consequently, the spiking control could be used to normalize for small fluctuations in DNA recovery. Twenty microliters of eluted DNA was added to 10 U of BstUI or HpaII (Fisher Scientific). Samples were incubated for 16 h at 37 °C followed by 20 min at 95 °C. The digested DNA sample was added 10× volume of DNA-free water (Sigma-Aldrich) and stored at −20 °C.

### Detection of hypermethylated *RASSF1A*

The detection of hypermethylated *RASSF1A* was based on the restriction enzymes HpaII and BstUI that cleaves unmethylated 5′-CCGG and 5′-CGCG motifs, respectively [29]. Notably, methylations of CpG in the cleavage motifs remain intact and detectable by real-time PCR. The PCR amplifications were done in duplicates of 20 μl each containing 250 pmol/L of primer using the Power SYBR Green PCR Master Kit (Life Technologies). The PCR was carried out on a 7500 Fast Real-Time PCR apparatus (Applied Biosystems). The inhibitory effect of interfering Mg^++^ from the Tango buffer on PCR amplification process was investigated. A final concentration of 3 % Tango buffer was found to have no influence on the efficiency of the PCR amplification. A primer set was designed against the spiking control to cover HpaII and BstUI cleavage motifs. This primer set was used to validate for 100 % cleavage efficiency of all DNA samples. A time-course study revealed that all PCR amplicons and the spiking control were 100 % cut after 2 h of incubation at 37 °C. As a standard, the incubation time was set to 16 h. Furthermore, a restriction-insensitive primer set was designed against a DNA region in the spiking control that did not contain HpaII and BstUI cleavage motifs. After PCR amplification of HpaII- or BstUI-pretreated DNA, the original serum concentration of hypermethylated *RASSF1A* was then expressed in ng/ml by using a DNA standard curve and with minor corrections for changes in DNA recovery.

### Availability of supporting data

None.

## Abbreviations

CA 15-3: cancer-antigen 15-3
CEA: carcinoembryonic antigen
TPA: tissue polypeptide antigen
*RASSF1A* gene: RAS association (RalGDS/AF-6) domain family member 1A
